# Whole-genome sequencing with long reads reveals complex structure and origin of structural variation in human genetic variations and somatic mutations in cancer

**DOI:** 10.1186/s13073-021-00883-1

**Published:** 2021-04-29

**Authors:** Akihiro Fujimoto, Jing Hao Wong, Yukiko Yoshii, Shintaro Akiyama, Azusa Tanaka, Hitomi Yagi, Daichi Shigemizu, Hidewaki Nakagawa, Masashi Mizokami, Mihoko Shimada

**Affiliations:** 1 Department of Human Genetics, Graduate School of Medicine, The University of Tokyo, Tokyo, Japan; 2 Department of Drug Discovery Medicine, Kyoto University Graduate School of Medicine, Kyoto, Japan; 3 Medical Genome Center, National Center for Geriatrics and Gerontology, Obu, Japan; 4 Laboratory for Cancer Genomics, RIKEN Center for Integrative Medical Science, Yokohama, Japan; 5 Genome Medical Sciences Project, National Center for Global Health and Medicine, Tokyo, Japan

**Keywords:** Long reads, Origin of structural variations (SVs), Germline SVs, Somatic SVs

## Abstract

**Background:**

Identification of germline variation and somatic mutations is a major issue in human genetics. However, due to the limitations of DNA sequencing technologies and computational algorithms, our understanding of genetic variation and somatic mutations is far from complete.

**Methods:**

In the present study, we performed whole-genome sequencing using long-read sequencing technology (Oxford Nanopore) for 11 Japanese liver cancers and matched normal samples which were previously sequenced for the International Cancer Genome Consortium (ICGC). We constructed an analysis pipeline for the long-read data and identified germline and somatic structural variations (SVs).

**Results:**

In polymorphic germline SVs, our analysis identified 8004 insertions, 6389 deletions, 27 inversions, and 32 intra-chromosomal translocations. By comparing to the chimpanzee genome, we correctly inferred events that caused insertions and deletions and found that most insertions were caused by transposons and *Alu* is the most predominant source, while other types of insertions, such as tandem duplications and processed pseudogenes, are rare. We inferred mechanisms of deletion generations and found that most non-allelic homolog recombination (NAHR) events were caused by recombination errors in SINEs. Analysis of somatic mutations in liver cancers showed that long reads could detect larger numbers of SVs than a previous short-read study and that mechanisms of cancer SV generation were different from that of germline deletions.

**Conclusions:**

Our analysis provides a comprehensive catalog of polymorphic and somatic SVs, as well as their possible causes. Our software are available at https://github.com/afujimoto/CAMPHOR and https://github.com/afujimoto/CAMPHORsomatic.

## Background

An abundance of genetic variation and somatic mutations exist within the human genome. Genetic variants are involved in disease risk and phenotypic variation among individuals [1]. Somatic mutations are known to cause cancerogenesis and rare diseases [2–4]. Identification of these variants and mutations is a crucial issue in human genetics. For this purpose, tremendous efforts have been made to create a comprehensive catalog of genetic variations and cancer somatic mutations [3, 5–7]. In the past decade, the application of next-generation sequencing technologies and the development of analysis algorithms have successfully identified variations and somatic mutations. However, due to the limitations of DNA sequencing technologies and computational algorithms, our understanding of genetic variations and somatic mutations is far from complete [8, 9].

In particular, identification of structural variations (SVs) is still difficult with current short-read methods. To overcome this problem, the development of data analysis methods and the application of long-read sequencing technologies have been conducted [10]. Recent studies have revealed that long reads are capable of identifying large numbers of SVs and complex SVs [11–13]. Long reads were also able to show the true structures of SVs and correct misclassification of pathogenicity [14]. Association studies between SVs and gene expression level showed that at least a few percentage of them influence gene expression level [15, 16], suggesting that SVs have functional importance and may explain a part of missing heritability in human disease. In cancer genome sequencing studies, analysis of SVs is also important to find driver genes and the mechanism of cancerogenesis [2, 3]. However, our understanding of somatic SVs is still incomplete, and the mechanism of SVs generation in somatic tissues remains elusive.

To obtain a comprehensive picture of germline variations and somatic mutations, and to infer the biological mechanism of SV generation, we performed whole-genome sequencing with a long-read sequencing technology (Oxford Nanopore). To compare the efficiency of long reads with that of short reads, we re-sequenced whole genomes of 11 Japanese liver cancer and matched normal samples that were previously analyzed in the International Cancer Genome Consortium (ICGC) [2, 3]. To detect polymorphic SVs and somatic SVs, we developed a computational analysis pipeline (named CAMPHOR). Inferring phylogenic status of germline variations clearly revealed sources of insertions and possible mechanisms of deletion generation. Analysis of somatic SVs in liver cancers showed that long-read technology could detect a larger number of somatic SVs and virus integrations. Comparison of the pattern of the SV breakpoints indicated differences between somatic mutations and germline variations. Our study reveals the advantages of long reads in analyzing human polymorphisms and somatic mutations.

## Methods

### DNA samples

We sequenced previously analyzed samples in the ICGC liver cancer project [2]. Eleven samples were selected and used for the sequencing. Informed consent to participate in the study was obtained from all subjects following the ICGC guidelines [2]. IRBs at RIKEN and Kyoto University and all groups participating in this study approved this work.

### Library preparation and sequencing

Libraries were prepared with the SQK-LSK108 library preparation kit (Oxford Nanopore). In brief, 2μg of DNA were fragmented using g-Tube (Covaris) by centrifugation at 10,000 rcf. End-prep and adapter ligation reactions were performed according to the manufacturer’s instructions. After purification, 15 μl library (~ 100 ng/μl) was used for sequencing. Sequencing was done using 106 flowcells with 48-h runs (Oxford Nanopore). Ten runs were performed for each sample. For samples which we could not obtain a sufficient amount of data, we performed additional runs. Base calling was done by albacore (Oxford Nanopore), and fastq files were obtained.

### Identification of germline SVs

We developed an analysis pipeline named CAMPHOR (comprehensive analysis method for polymorphic and somatic structural variations). Mapping was done by minimap2 [17] software with the following option: “-a -g2000 -A1 -B2 -O2,32 -E1,0 -z200,” and ≥ 500-bp unmapped region (soft-clipping regions and unmapped reads) was mapped by bwa [18] (version0.7.12) with the following option: “mem -x ont2d.” We removed alternative contigs from GRCh38 and used it as the reference genome sequence. First, we identified deletions (≥ 100 bp), insertions (≥ 100 bp), inversions, and intra-chromosomal translocations. The expected patterns of support reads are shown in Fig. S1 (Additional file 1). Expected patterns of insertions are classified into two types: insertions within reads and a cluster of reads with unmapped regions (Additional file 1: Fig. S1). We first clustered nearby SV supporting reads (within 50 bp range for deletions and insertions, 100 bp for inversions and translocations), and SVs supported by ≥2 reads with mapping quality ≥20 were considered as candidates. The identified SV breakpoints were merged if they were within 300 bp (insertion) or 1000 bp (others) and 80% of SV regions overlapped. We removed reads that detected SVs within the edges of reads (10% of length). For deletions, we observed that insertions occurred close to deletions, and this caused false-positive deletions; therefore, we removed the deletion reads if deletions have insertion with length ≥ 10% (for < 1000 bp deletions) or 30% (for ≥1000 bp deletions) of deletion length within 30-bp regions from the breakpoints.

High deletion error rates exist in Nanopore sequencers, and shorter indel errors were predominant. Therefore, we considered 100–500 bp deletions supported by ≥4 reads and 501–1000 bp deletions supported by ≥3 reads for further analysis. For insertions, we considered ≤1000 bp insertions supported by ≥3 reads containing insertions and > 1000 bp insertions supported ≥2 reads containing insertions or ≥ 2 soft clipped reads at both sides (Additional file 1: Fig. S1).

We then applied a filter for germline SVs. We removed SVs within regions with many unreliably mapped reads (≥ 30% reads having mapping quality < 30) and SVs with variant allele frequency < 0.03. For inversions and translocations, we removed candidates if ≥80% of the region was covered by short repeats detected by RepeatMasker [19] and Tandem Repeat Finder [20]. For inversions and translocations, we also removed candidates that had both breakpoints in different segments of the same segmental duplication (data of segmental duplications were obtained from the UCSC Genome Browser). False-positive SVs can be caused by artificial chimeric reads, and our program tried to remove them with read information (Additional file 1). Details of the SV calling are described on the CAMPHOR website.

The identified germline SVs from each sample were merged, and SVs with allele frequencies in 11 samples ≥0.1 were selected.

### Identification of somatic SVs

Somatic SVs were identified by comparing SVs detected from cancer with those from matched normal samples. We detected SVs as mentioned in *Identification of germline SVs*. For somatic SVs, we additionally identified chromosomal translocations. The identified SV breakpoints of chromosomal translocations were merged if they were within 500 bp.

We then applied filtering for somatic SVs. We merged germline SVs of all samples and used the resultant as a “normal panel”. We removed SVs if they were detected in the normal panel or if the depth of coverage in the matched normal sample was < 9. We also removed SVs within regions with many unreliably mapped reads (≥ 30% reads having mapping quality < 30), as well as SVs with variant allele frequency < 0.03. We also removed candidates with both breakpoints in different segments of the same segmental duplication (data of segmental duplications was obtained from the UCSC Genome Browser). For deletions, translocations, and inversions, we removed candidates if ≥80% of the region was covered by short repeats detected by RepeatMasker [19] and Tandem Repeat Finder [20]. False-positive SVs can be caused by artificial chimeric reads, and our program tried to remove them with read information (Additional file 1). Details of the SV calling are described in the CAMPHOR website. For all candidates, we manually reviewed the somatic SV candidates, and if other types of SVs are observed in the matched normal samples, these were removed. This manual review removed about 10% of the somatic SV candidates.

### Validation of SV call

To evaluate the sensitivity of our analysis, we compared the results of SV calling from Nanopore with those from short reads [2]. For the comparison, we lifted genomic coordinates of SVs in the previous study from GRCh37 to GRCh38 with the liftOver software. Specificity was evaluated with PCR. For PCR, we added Betaine solution (SIGMA) to the PCR reaction mix, which dissolves the secondary structure of DNA. For some of the SV candidates, we used several PCR primer sets or the nested-PCR method.

### Generation of consensus sequences

Since the error rate of the Nanopore sequencer was not insignificant, we generated consensus sequences for germline insertions and deletions and for somatic SVs. We first gathered all SV support reads. In germline SVs, we gathered SVs support reads from all samples having the same SVs. From each read, we extracted the sequence of indels and their flanking regions (±500 bp from the breakpoints) and aligned them with the MAFFT software [21] with the following options: “--retree 2 --maxiterate 2” for candidates with a number of reads > 1000 or maximum length of region > 2000, or “--ep 0.0 --op 1 --maxiterate 1000 --globalpair” for others. Based on the multiple alignments, we assumed major bases (≥ 50% at each position) as true bases and generated consensus sequences.

We then mapped the consensus sequences to the reference genome with the BLAT software with the following option: “-tileSize=9 -stepSize=5 -minMatch=2 -minScore=10 -minIdentity=70 -maxGap=2 -repMatch=2253.” For deletions, we removed the consensus sequences if the mapped locations were not overlapped with the original calls. For insertions, we removed the consensus sequences if distances between original locations and BLAT mapping locations were larger than 100 bp.

To identify repeats in each consensus sequence, we used RepeatMasker [19] and Tandem Repeat Finder [20] with options (RepeatMasker: “-a -xsmall” and Tandem Repeat Finder: “2 7 7 80 10 50 500 -f -d -m”).

To identify past events that caused insertions and deletions, we inferred the ancestral status for them. We converted locations of ±100 bp regions from breakpoints of indels in GRCh38 build to those in PanTro6 (Chimpanzee) and calculated the ratio (distance between breakpoints in PanTro6 - 200 bp)/(distance between breakpoints in GRCh38 - 200 bp). For deletions, we consider that chimpanzee has deletions if the ratio was − 0.3–0.3, and not if it was 0.7–1.3. For insertions, we consider that chimpanzee has insertions if the ratio was 0.7–1.3, and not if it was − 0.3–0.3.

We performed the same procedure for generating consensus sequences for somatic SVs.

### Identification of virus integration events

We mapped all reads to hepatitis B virus (HBV) and adeno-associated virus (AAV) genome sequences. We mapped all reads to multiple virus reference genomes as performed in previous studies [2, 22] and selected the reference genome with the largest number of mapped reads as the best reference for each sample. Then, reads mapped to the best reference virus genome and the human reference genome were used to identify virus integration sites.

### Analysis of features of SVs

To find the factors that influence the mutability of SVs, we considered replication timing and chromatin state. For replication timing, we used data of several cells as performed in a previous study [23]. The genomic locations of the replication timings were based on GRCh37; therefore, we lifted them to GRCh38 with the liftOver software. For the analysis of the chromatin state, we focused on the regions within 100 bp from breakpoints. To obtain the expected numbers of 100-bp bins overlapping each chromatin state, we randomly selected 500,000 bins from the reference genomes and calculated the proportions of bins overlapping each chromatin state. The proportions of each chromatin state were Het 0.0288, Quies 0.673, TssA 0.0115, TxWk 0.160, ZNF/Rpts 0.00279, Enh 0.0426, TssAFlnk 0.00300, ReprPCWk 0.0428, ReprPC 0.0108, EnhBiv 0.00229, TssBiv 0.00477, BivFlnk 0.000758, Tx 0.0157, EnhG 0.00115, and TxFlnk 0.000389. The expected numbers were calculated for each chromatin state and used for Fisher’s exact test.

### Benchmarking

We compared the accuracy of our tool with previously published methods. We analyzed the whole-genome sequencing data of NA19240 released by a previous study [13]. Since the data size of NA19240 (~90×) is much larger than that of the current study and standard whole-genome sequencing studies, we selected one read from every 5 reads in the data and used these for the benchmarking. We mapped the data to the reference genome sequence with minimap2 and detected SVs with SVIM (v1.4.2) and sniffles (v1.0.12) [24, 25] with default settings. Since our analysis focused on indels with ≥100 bp, we selected indels with ≥100 bp and used these for the benchmarking. We additionally performed SV selection for SVIM. The output of SVIM reported quality score for all SVs, and De Coster et al. selected SVs with a quality score of ≥40 [13]. However, in this benchmarking, filtering with a quality score of ≥ 40 removed most of all SVs and was considered too conservative. Therefore, we selected SVs with various quality scores (≥ 0 (all SVs), ≥ 5, ≥ 10, ≥ 20, ≥ 30, and ≥ 40 (used in De Coster et al)).

Since most of all SVs are indels and nomenclatures of other SVs are inconsistent among callers, we only evaluated indels in the benchmarking. We also classified SVs with repeat information. Since tandem repeats are unstable and self-chain regions cause false-positive calls due to mapping errors, SVs in tandem repeat regions, self-chain regions, regions in both repeats, and non-repeat regions were evaluated separately.

The results of SV calling were compared with gold standard SV calls as used in De Coster et al. We considered indels as commonly identified if both were the same type, and distance between breakpoints of identified SVs and those of the gold-standard SV calls < 500 bp [13]. Based on the comparison, the SVs were classified into three groups (common: detected in gold standard SV calls and each caller; gold standard SV call only: detected only in gold standard SV calls; and caller only: detected only in each caller), and we compared the numbers. We also evaluated *F*-measures. For this evaluation, SVs in “gold standard SV call only” were considered as false negatives and “caller only” as false positives (gold standard SV should not be perfect and may contain false-positive calls, but in this comparison, we used this classification for simplicity).

### Analysis of methylation rate

We analyzed methylation rate using tombo (version: 1.5.1) according to the instructions. For the analysis, we selected the promoter of *ALB* and *TERT* genes for methylation analysis. We extracted reads mapped to ±10kbp from the transcription start site of *ALB* and *TERT* genes. Since the depth of coverage is not high in each sample, we merged all reads and compared the methylation rates between blood and liver cancer for *ALB* and liver cancers with and without *TERT* mutations. Mutation information in *TERT* was obtained from our previous study [2].

## Results

### Samples and sequencing

We performed whole-genome sequencing of 11 liver cancer and matched normal pairs with MinION (Oxford Nanopore). All of them have been sequenced by a short-read sequencer (Illumina) and reported in previous papers [2, 3] (Additional file 2: Table S1). Samples with ≥60 μg genomic DNA available were selected for the whole genome sequencing. Ten runs were performed for each sample, and an average of 53.9Gbp of sequence data was obtained (Additional file 2: Table S2). The maximum read length was 495kbp, and the average read length was 5457.9 bp (Additional file 2: Table S3, Additional file 1: Fig. S2a).

Mapping was performed using the minimap2 software to the human reference genome (GRCh38) [17]. The average mapping rate (number of mapped reads/total number of reads) and proportion of aligned bases to total bases (total aligned bases/total bases of reads) were 83.7% and 89.7%, respectively, suggesting that about 90% of the data was aligned to the reference genome sequence (Additional file 2: Table S3). Unmapped reads had lower average base quality (Additional file 1: Fig. S2b). In the mapped reads, most of all regions were mapped (Additional file 1: Fig. S2c), and their mapping qualities, which were generated by minimap2 as the measure of mapping uniqueness, were high (Additional file 1: Fig. S2d). Average edit distance, insertion rates, and deletion rates were 14.8%, 4.4%, and 6.6%, respectively, which is consistent with a recent study [26] (Additional file 1: Fig. S2e). These results showed that the error rates of Nanopore long reads were not low, but the majority of reads were uniquely mapped to the human reference genome and could be used for the identification of structural variations (SVs).

### Identification of germline structural variations

We first analyzed normal samples to detect germline polymorphic SVs. We detected deletions (≥ 100 bp), insertions (≥ 100 bp), inversions, and intra-chromosomal translocations (see the “Methods” section). Our analysis method identified 17,582 insertions, 12,249 deletions, 107 inversions, and 265 intra-chromosomal translocations (Additional file 2: Table S4). To make our analysis conservative, we selected germline SVs found with allele frequencies ≥0.1 (≥ 3 in 22 alleles). Our analysis detected 8004 insertions, 6389 deletions, 27 inversions, and 32 intra-chromosomal translocations (Additional file 2: Table S5, 6). Most inversions and intra-chromosomal translocations were short, and all intra-chromosomal translocations were considered to be tandem duplications (Additional file 2: Table S6). The distribution of indel (insertion and deletion) length showed that the majority of them were short, and secondary peaks around ~ 300 bp were found in both insertions and deletions, as previously reported (Additional file 1: Fig. S3a) [13]. We then compared the population frequencies of recurrent insertions and deletions in different functional categories. As expected, deletions overlapping coding regions were significantly rarer than other deletions (*p* value = 3.5 × 10^−5^, Fisher’s exact test), suggesting that they have slightly deleterious effects [27] (Additional file 1: Fig. S3b, Additional file 2: Table S7).

### Benchmarking

**Fig. 1:**
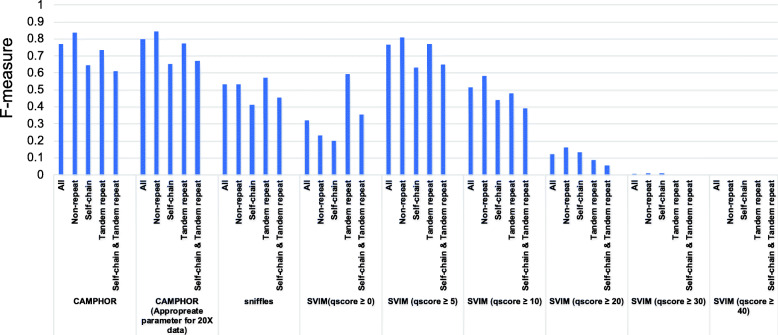
Result of benchmarking using NA19240. Indels (≥ 100 bp) were identified with several software from the fastq file of NA19240 and compared with a gold standard SV callset as performed previously [13]. We classified SVs as common; detected in gold standard SV calls and each caller, gold standard SV call only; detected only in gold standard SV calls and caller only; detected only in each caller. SVs in “gold standard SV call only” were considered false negatives and “caller only” false positives, and *F*-measures were calculated with sensitivity and specificity. SVs were classified based on repeat information of regions. SVs in tandem repeat regions, self-chain regions, regions in both repeats, and non-repeat regions were evaluated separately. “CAMPHOR” was a result of parameter setting in this study, and “CAMPHOR (appropriate parameter set for 20× data)” was a result of the released version

To compare the accuracy of our SV caller, we performed SV calling using previously sequenced data of NA19240 [13]. We detected SVs with SVIM, sniffles, and CAMPHOR (our tool) and compared the results with a “gold standard set of SVs” [13, 24, 25] (Additional file 2: Table S8, Additional file 1: Fig. S4). In this comparison, sniffles was conservative (smaller number of “common” and “caller only”). Among the different quality score thresholds, SVIM (quality score ≥ 0 (all SVs)) was too progressive (larger number of “common” and “caller only”) while SVIM (quality score ≥ 40) was too conservative (very small number of “common” and “caller only”) (Additional file 1: Fig. S4). SVIM (quality score ≥ 5) showed a good balance and the highest *F*-measure (Fig. 1, Additional file 1: Fig. S4). Our tool (CAMPHOR) showed better performance than sniffles and SVIM with various quality score filters and showed similar (slightly better) performance against SVIM (quality score ≥ 5) (*F*-measure 0.770 (CAMPHOR) vs 0.765 (SVIM (quality score ≥ 5))) (Fig. 1, Additional file 1: Fig. S4). These results suggest that our tool has good accuracy for analyzing SVs.

### Inference of ancestral events and possible causes of germline indels

We aimed to detect the causes of germline indels (Additional file 1: Fig. S5)*.* Since the error rate of the Nanopore sequencer was not insignificant (Additional file 1: Fig. S2e), we needed to construct accurate sequences of insertions and breakpoints of deletions. To construct consensus sequences from reads, we extracted the sequence of indels and their flanking regions (±500 bp from the breakpoints) and aligned them with the MAFFT software [21]. Based on the multiple alignments, we generated consensus sequences for insertions and deletions. From the 8004 recurrent insertions and the 6389 recurrent deletions, consensus sequences were obtained for 7924 insertions and 6389 deletions. We then mapped the consensus sequences to the human reference genome sequence using the BLAT software [28], and consensus sequences of 6433 insertions and 5622 deletions were properly mapped. We then analyzed sequences with the RepeatMasker [19] and Tandem Repeat Finder [20] software and removed sequences ≥50% covered by short repeats (Additional file 1: Fig. S6), as they cannot be used for the subsequent analysis (see Discussion). As a result, we obtained consensus sequences of 6953 non-short repeat indels (3709 insertions and 3224 deletions) (Additional file 1: Fig. S5).

**Fig. 2:**
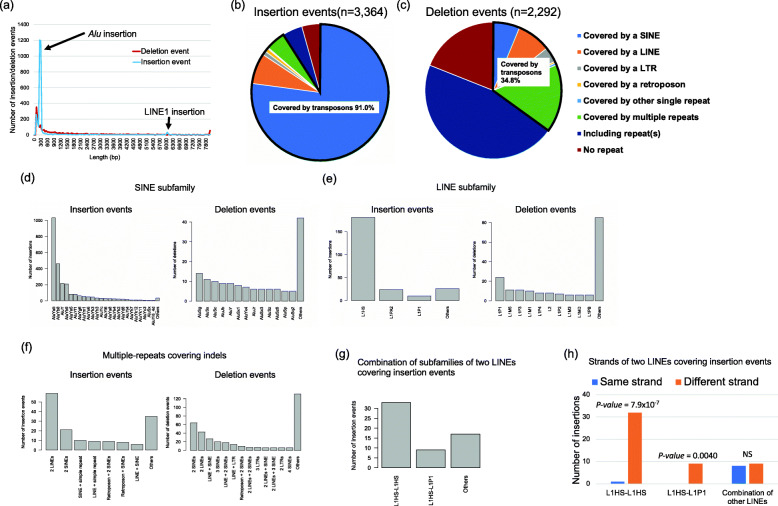
Germline insertions and deletions. **a** Length distribution of insertion and deletion events. Insertions and deletions with length ≥ 100 bp were identified, classified to insertion and deletion events, and lengths were compared. **b** Classification of the repeat(s) in insertion events. **c** Classification of the repeat(s) in deletion events. We considered sequences as covered by repeat(s) if repeat(s) occupied ≥80% of the sequences. **d** Classification of SINE families in insertion and deletion events. Families of SINEs that covered indel regions were analyzed. Families with count ≤5 were classified as others. **e** Classification of LINE families in insertion and deletion events. Families of LINEs that covered the indel regions were analyzed. Families with count ≤5 were classified as others. **f** Composition of multi-repeats covering indels. Families with count ≤5 were classified as others. **g** Combination of LINE subfamilies of two LINEs covering insertion events. **h** Strands of the combination of two LINEs found in insertion events. Combinations of different strands were significantly larger than that of the same strands in L1HS-L1HS and L1HS-L1P1 (Fisher’s exact test)

Next, we inferred ancestral events for the indels according to the phylogenic status of indels. Our indel detection is dependent on the reference genome sequence; therefore, insertions in the reference genome samples should be detected as deletions and vice versa. To infer the cause of indels, we had to define events that generated indels, rather than reference genome-based indel status (Additional file 1: Fig. S7). For this purpose, we compared the indel regions with the chimpanzee genome. We considered an insertion candidate as “an insertion caused by a deletion event in the human population” if it was present in the chimpanzee genome and “an insertion caused by an insertion event in the human population” if not seen in the chimpanzee genome (Additional file 1: Fig. S7). We also considered a deletion candidate as “a deletion caused by an insertion event in the human population” if it was present in the chimpanzee genome and “a deletion caused by a deletion event in the human population” if not seen in the chimpanzee genome (Additional file 1: Fig. S7). As a result of this comparison, we obtained 3364 insertion and 2292 deletion events (1297 were unable to be classified). The length distributions of the events were quite different from those of the reference genome-based indels. The peak at 300 bp in deletions was absent from the deletion events (Fig. 2a and Additional file 1: Fig. S3a). For insertion events, a very clear peak at 300 bp and a small peak at 6kbp were observed, which could be explained by *Alu* and LINE1 insertions (see below).

### Analysis of the features of germline insertion and deletion events

Repetitive elements can cause insertion and deletions; therefore, we first focused on the repeat features of inserted and deleted sequences using the RepeatMasker [19] software. We considered sequences as covered by repeat(s) if repeat(s) occupied ≥80% of the sequences. Patterns of repeats were quite different between insertion and deletion events (Fig. 2b, c and Additional file 2: Table S9). Among the inserted sequences, 91.0% are involved in transposable elements, and single SINE and LINE account for 84.3% of the insertion events (Fig. 2b and Additional file 2: Table S9). About 77.1% of insertion events were covered by a single SINE, followed by 7.2% by a single LINE, 4.7% by multiple repeats, 4.7% included repeats, and 4.3% of non-repeats (Fig. 2b and Additional file 2: Table S9). In contrast, 34.8% of deletion events were covered by transposable elements, and only 6.3% and 8.1% of the deletions were covered by a single SINE or LINE, respectively (Fig. 2c and Additional file 2: Table S9).

We further analyzed sub-families of SINEs and LINEs. In the insertion events, *AluYa5*, *AluYb8*, *AluY*, and *AluYb9* were predominant, which were reported as active SINEs in a previous experimental study [29] (Fig. 2d). Regarding LINEs, the majority belonged to the L1HS subfamily, which is also known as an active LINE [30] (Fig. 2e). In contrast, there were no predominant subfamilies in the deletion events (Fig. 2d, e). The analysis of the inserted regions of SINEs and LINEs suggests that a part of SINEs and more than half of LINEs lacked 5′ regions (Additional file 1: Fig. S8). Insertion of SINEs and LINEs started from their 3′ ends [31], and their integration would be sometimes stopped or inhibited during integration.

Our analysis also identified insertions and deletions covered by multiple repeats. The patterns were also different between the insertion and deletion events. In insertion events, the combination of 2 LINEs was predominant (Fig. 2f), and most of them were combinations of L1HS-L1HS or L1HS-L1P1 subfamilies (Fig. 2g). Analysis of DNA strands showed that they had significantly higher proportions of different strands (L1HS-L1HS; *p* value = 7.9 × 10^−7^, L1HS-L1P1; *p* value = 0.0040, Fisher’s exact test) (Fig. 2h and Additional file 2: Table S10). This pattern can be explained by a previously proposed LINE integration model (twin priming model) [32]. Of the 242 single LINE and 59 two LINE insertions, 50 (16.6%) could be explained by the twin priming model (Additional file 2: Table S9, 10). We also performed the same analysis for SINE insertions and found that they had a significantly higher proportion of the same strands (*p* value = 0.0015, Fisher’s exact test) (Additional file 1: Fig. S9 and Additional file 2: Table S11).

**Fig. 3:**
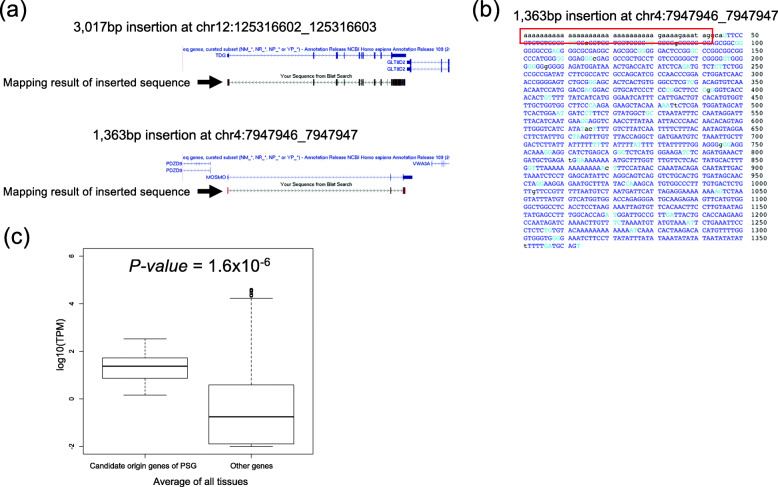
Polymorphic insertion of processed pseudogenes. Analysis of long reads revealed the entire structures of insertion of processed pseudogenes. **a** Examples of the processed pseudogene. Inserted sequences of 3017 bp insertion at chr12:125316602_125316603 and 1363 bp insertion at chr4:7947946_7947947 were mapped to exons of *TDG* and *MOSMO* genes. **b** An example of non-reference poly(A) sequence in a polymorphic insertion of processed pseudogenes. An output of web-BLAT is shown. Blue and black characters indicate aligned and unaligned bases, respectively. Black characters in the sequences show the nucleotides that are not found in the reference genome sequence. **c** Average expression level of candidate origin genes of processed pseudogenes. The expression data was obtained from GTEx expression data [33], and the average expression level was calculated. The expression levels were compared between the candidate origins and other genes (Wilcoxon signed-rank test)

Only 4.3% (*n* = 146) of the insertions were not related to the repeat elements. To know the origin of the inserted sequences, we mapped the sequences to the human reference genome with the BLAT software [28]. Of these, 74 and 15 were mapped to the reference genome and were considered to be tandem duplications and template sequence insertions (Additional file 2: Table S12). Fifteen inserted sequences were mapped to exonic regions (Fig. 3a and Additional file 2: Table S13). Six of them were split and mapped to exons (Fig. 3a), and the others were mapped to 3′ regions of genes (Additional file 2: Table S13). Among them, 10 had poly(A) or poly(T) at the end of sequences that were not found in the reference genome (Fig. 3b).

These results suggest that the source of these insertions were spliced exons, and reverse-transcribed mRNAs were inserted into the genome sequences (processed-pseudogenes) [34]. We then compared the gene expression level of the candidate source genes in 54 tissues (Fig. 3c, Additional file 1: Fig. S10 and Additional file 2: Table S14). These 15 genes showed significantly higher expression level than other genes in all tissues (*p* value = 1.6 × 10^−6^, Wilcoxon signed-rank test), suggesting that ubiquitously expressed genes can be origins of processed pseudogenes [34, 35] (Fig. 3c, Additional file 1: Fig. S10 and Additional file 2: Table S14).

### Causes of germline deletion events

**Fig. 4:**
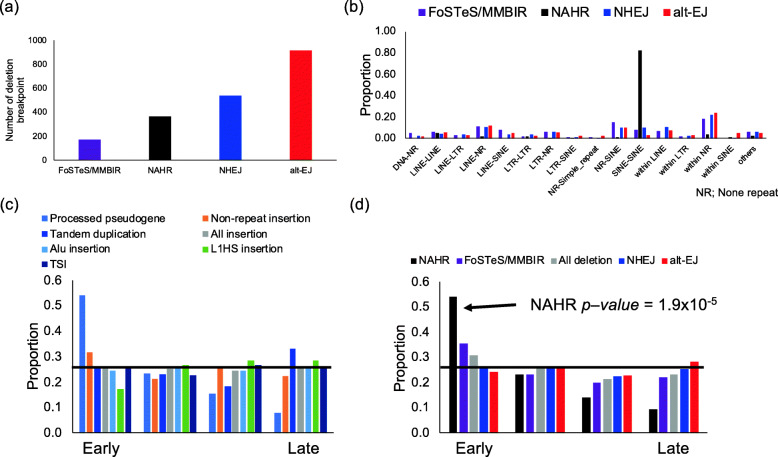
Analysis of the mechanism of germline insertion and deletion events. **a** Possible mechanism of germline deletion events. The mechanism was estimated from the flow in Fig. S12 (Additional file 1). **b** Analysis of repeat of breakpoints of each deletion mechanism. Combinations of repeats at both breakpoints are shown. NR, non-repeat. “Within” indicates both breakpoints were included in the same repeats or non-repeats. **c** Replication timing and insertion events. **d** Replication timing and deletion events. Replication timing is represented by four groups from left to right, earliest to latest. The proportions of each category are represented by colored bars. The black line is at 0.25, the proportion expected in each group if the categories were divided equally among them. NAHRs were biased toward early replicating regions

Deletions are known to be generated by several mechanisms, non-homologous end joining (NHEJ), alternative end joining (alt-EJ), non-allelic homologous recombination (NAHR), and folk stalling and template switching or microhomology-mediated break-induced repair (FoSTeS/MMBIR) [36, 37]. Previous studies inferred the causes of deletions by analyzing the structures of breakpoints [36, 37]. In an attempt to elucidate the mechanism of deletion generation, we analyzed the sequences of deletion breakpoints as performed in a previous study [37]. Reference genome sequences covering upstream and downstream breakpoints were aligned to consensus sequences, and we analyzed the homology length and insertion between breakpoints (Additional file 1: Fig. S11, S12a) [36, 37]. Among the 2292 deletion events, our analysis estimated there to be 363 events caused by NAHR, 916 by alt-EJ, 172 by FoSTeS/MMBIR, and 537 by NHEJ (Fig. 4a). The proportions of alt-EJ, FoSTeS/MMBIR, and NHEJ were consistent with those in a previous study (Additional file 1: Fig. S12b) [37]. In addition to the previous study, our long-read analysis identified deletions caused by NAHR, which was defined with > 100 bp homology and would be difficult to be identified by short reads. A comparison of deletion sizes caused by each mechanism showed that deletions caused by FoSTeS/MMBIR and NHEJ were significantly larger than those by NAHR and alt-EJ (Additional file 1: Fig. S13).

Deletions with insertions between their breakpoints were classified as NHEJ or FoSTeS/MMBIR based on the insertion length (Additional file 1: Fig. S12a). We analyzed the pattern of the insertions. Deletions classified as NHEJ can contain short insertions (Additional file 1: Fig. S12a). In our analysis, shorter insertions were larger (Additional file 1: Fig. S14a), and the pattern of nucleotides did not skew from randomness (Additional file 1: Fig. S14b). In the insertions of FoSTeS/MMBIR candidates, the number of shorter sequences was larger, but longer sequences were inserted as well (Additional file 1: Fig. S15a). We performed PCR and Sanger sequencing of three candidates and confirmed the presence of the insertions at the breakpoints (Additional file 1: Fig. S15b). To find their source, we mapped 73 insertions with ≥30 bp to the reference genome with the BLAT software [28]. Thirty-three of them were mapped to the reference genome (Additional file 2: Table S15).

We then analyzed the features of the breakpoints of each type of the deletions. We counted the combinations of repeats and found that more than 80% of the NAHR breakpoints were in SINEs (Fig. 4b). The locations of both breakpoints within SINEs corresponded (Additional file 1: Fig. S16). These results suggest that the majority of NAHRs were caused by recombination errors within SINEs.

### Factors that influence insertions and deletions

To examine the factors that influence insertions and deletions, we analyzed the association with replication timing, which is associated with mutation rates of SNVs, SVs, and microsatellites [2, 23, 38] (Fig. 4c, d). As shown in a previous study [39], deletion by NAHR was predominant in the early replicating regions (Fig. 4d). Insertions of processed pseudogenes were larger in the early replicating regions, but not statistically significant, possibly due to the small number of events (Fig. 4c). We also tested the association with chromatin states [40]. *Alu* and L1HS insertions were significantly enriched in the quiescent state regions, and *Alu* insertions were significantly underrepresented in the enhancer regions (*Alu* in a quiescent state, *p* value = 9.3 × 10^−6^; L1HS in a quiescent state, *p* value = 6.5 × 10^−4^; and *Alu* in an enhancer state, *p* value = 8.2 × 10^−5^, Fisher’s exact test) (Additional file 2: Table S16). In the deletions, NAHR and alt-EJ were significantly enriched in quiescent state and actively transcribed states, respectively (NAHR in a weak actively transcribed state, *p* value = 1.3 × 10^−5^; alt-EJ in a quiescent state, *p* value = 3.2 × 10^−5^) (Additional file 2: Table S17).

### Identification of somatic SVs in liver cancers

We then identified somatic SVs by comparing cancer and matched normal samples. In this study, we identified deletions (≥ 100 bp), insertions (≥ 100 bp), inversions, and intra- and inter-chromosomal translocations as well as integrations of HBV. In total, our analysis detected somatic 919 SVs (278 deletions, 205 intra-chromosomal translocations, 48 insertions, 230 inversions, and 158 inter-chromosomal translocations) and 26 HBV integrations (Additional file 2: Table S18, 19).

**Fig. 5:**
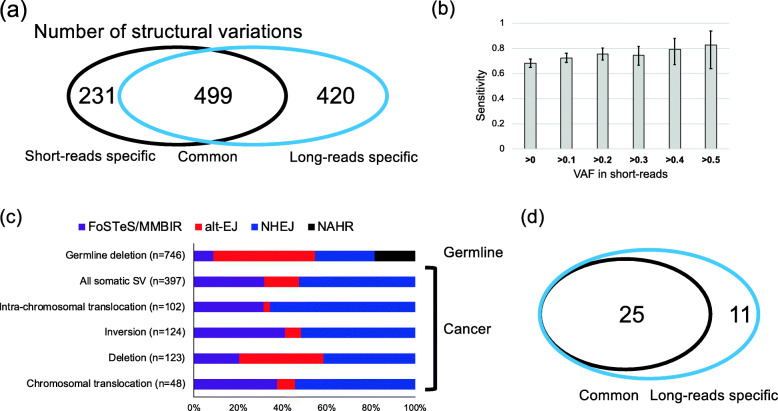
Somatic SVs in liver cancers. **a** Comparison of somatic SVs between short-read study and the current study. Somatic SVs detected by this study were compared with a previous short-read study [2]. **b** Sensitivity of SV calling in the current study. We calculated the sensitivity for SVs identified by short reads. Variant allele frequencies (VAFs) were estimated from short reads [2]. **c** Possible mechanism of somatic SVs. The mechanism was estimated as done for germline deletions. The proportions were significantly different between germline deletions and somatic SVs (*p* value = 9.9 × 10^−77^, chi-square test). **d** Comparison of HBV integrations between short-read study and this study. HBV integrations identified by this study were compared with a previous short-read study [2]

We compared the SV candidates with SVs identified by a previous short-read study [2] (Fig. 5a). Of these, 499 SVs were commonly identified, and 231 and 420 SVs were identified only by short reads and long reads, respectively (Fig. 5a). The long reads identified 68.4% of SVs detected by short reads. The comparison of the variant allele frequency (VAF) of SVs showed that the undetected SVs had significantly lower VAFs (Additional file 1: Fig. S17a). We estimated the sensitivity of SV detection with various VAFs. As expected, SVs with higher VAF had higher sensitivities, and 80% of SVs with VAF ≥ 0.4 were detected by the current study (Fig. 5b). The VAFs were highly correlated between the short reads and long reads (Additional file 1: Fig. S17b), and the number of SVs by short reads were strongly correlated with that of long reads (Additional file 1: Fig. S17c). We manually reviewed the pattern of long reads at the breakpoint locations of the unidentified SVs with short reads VAF ≥ 0.3, and we could find one or no SV support read. These results suggest that false negatives in the current study were mainly due to low depth of coverage and not due to problems in long reads nor our analysis method. Higher depth should increase sensitivity.

To assess the false-positive rates of this analysis, we selected 115 SVs and performed validation with PCR. In the experiment, PCR amplification was more difficult than that for short-read SVs. Long reads may detect SVs in regions that are difficult to amplify by PCR. The addition of betaine to the PCR reaction mix was required, which dissolves the secondary structure of DNA. For some of the SV candidates, we tried to use several PCR primer sets or the nested-PCR method. Of the 115 SV candidates, 108 were successfully validated (false discovery rate = 7%). For the long read-specific candidates, 65 out of 72 candidates were validated (false discovery rate = 9.7%). These results suggest that our analysis method has sufficient accuracy for cancer genome analysis.

We then compared the repeat features of SVs between common and long read-specific SVs. SVs whose breakpoints were in any types of repeats were significantly enriched in the long read-specific SVs (*p* value = 4.4 × 10^−5^, OR = 2.0, Fisher’s exact test). In each repeat, LINEs were significantly enriched (LINE/LINE: *p* value = 2.5 × 10^−4^, OR = 3.2; LINE/-, *p* value = 0.044, OR = 1.8) (Additional file 2: Table S20). This result shows that long-read sequencing has an advantage in identifying repeat-mediated SVs.

### Causes of somatic SVs in cancer

We compared the features of breakpoints of somatic SVs as was done for the germline deletions (Additional file 1: Fig. S11, S12). We collected reads that cover the breakpoints and made a consensus sequence for them. Although the number of reads was not sufficient for all SVs, consensus sequences were obtained for 397 SVs. We inferred the SV generation mechanism from the breakpoint sequences. The proportions of each type were significantly different between the somatic SVs and the germline deletions (*p* value = 9.9 × 10^−77^, chi-square test) (Fig. 5c, Additional file 2: Table S21). Unlike the germline deletions, NAHR event, which is characterized by > 100 bp homology, was not detected in the somatic SVs, and the proportion of alt-EJ was larger than the germline deletions (Fig. 5c, Additional file 2: Table S21). We then analyzed cancer somatic SVs and compared each somatic SV type with all other SVs. Although the proportions of most SVs were not significantly different, the proportion of alt-EJ was significantly higher in somatic deletions (Fig. 5c, Additional file 2: Table S22).

One important feature of cancer SVs is accumulation in certain genomic regions, and the mechanisms underlying clustered SVs have been discussed [41]. We analyzed the breakpoints of SVs for clustered SVs with long reads. We collected reads with ≥2 breakpoints, and 21 reads were obtained. This analysis showed that some distant SVs in the reference genome were located close to each other, and long reads can show their true structures (Additional file 1: Fig. S18). The signature of NHEJ, alt-EJ, and FoSTeS/MMBIR were found in the breakpoints of clustered SVs (Additional file 2: Table S23).

### Virus integrations

We mapped all reads to hepatitis B virus (HBV) and adeno-associated virus (AAV) genome sequences, which were integrated into liver cancer genomes [2, 42]. No AAV genome sequence was detected. Reads mapped to the HBV genome were detected from four HBV-positive samples (RK014, RK020, RK085, and RK147) (Additional file 2: Table S24). Thirty-four integrations were identified, and 25 of them were found by short reads (Fig. 5d, Additional file 2: Table S25) [2]. Our analysis identified an integration to centromeric regions and an integration to SINE, suggesting that HBV can integrate to repetitive regions (Additional file 2: Table S25). Our analysis also detected full-length integration event to the *MLL4* (*KMT2B*) gene (Additional file 1: Fig. S19).

### Cancer SVs and genes

We searched SVs in the genic regions, and our analysis identified genes with recurrent breakpoints. Twenty-four genes had breakpoints in two samples (Additional file 2: Table S26). Next, we focused on previously suggested driver genes [43]. Within the 299 driver gene candidates, 9 driver genes (*MSH3*, *GNAQ*, *TCF7L2*, *MET*, *PDS5B*, *ARID2*, *PTCH1*, *NUP93*, and *ARID1A*) had SV breakpoints. Of these, five were previously identified by the short-read analysis [2]. In addition to these genes, our analysis identified seven SVs including in the *CDKN2A* gene, and none of them was detected by the previous short-read study [2]. The current study identified a new chromosomal translocation of the *TERT* upstream region whose breakpoints were in SINE (Additional file 2: Table S19). Eight SVs were detected in the *MACROD2* gene region in two samples, which is known as a fragile site in cancer.

### Analysis of methylation in TERT prompter

As methylation analysis is an important advantage of Nanopore sequencing technology, we additionally analyzed the methylation of the *TERT* promoter. *TERT* is an important driver gene in many cancers, and a previous study suggests that *TERT* is overexpressed by point mutations, SVs, copy number alternations, and HBV integrations [2]. To analyze the impact of mutations on methylation, we gathered reads from *TERT* promoter from samples with and without mutations. Although the methylation rates were not significantly different around the transcription start site and in the previously described *TERT* hypermethylated oncological region (THOR) [44], samples with mutated promoters had significantly lower methylation rates in their upstream and downstream regions (Additional file 1: Fig. S20).

## Discussion

Long-read technologies are expected to detect larger numbers of SVs and haplotype structures; however, due to high error rates, their efficacy for somatic mutations and germline variants is still controversial. In the present study, we analyzed the whole genomes of 11 cancer samples previously reported by ICGC [2, 3], which enabled us to evaluate the mutation calling with long reads (Fig. 5). Our analysis showed the advantage of long-read sequencing technologies. First, because of the longer read lengths, most reads were uniquely mapped to the human genome (with high mapping quality), and larger proportions of reads could be used for variant calling (Additional file 1: Fig. S2). Second, reads are longer than repeat elements, and SVs mediated by repeats can be detected (Additional file 2: Table S20). Indeed, in spite of the lower depth of coverage (long reads 17× vs. short reads ~40×), our analysis detected 1.6 times larger numbers of somatic SVs (Fig. 5). We also detected germline deletions by NAHR, which were difficult to be detected using short reads [37]. Third, entire sequences of insertions could be analyzed, which allowed us to detect structures of inserted transposable elements and processed pseudogenes (Figs. 2 and 3). Fourth, we could observe haplotype structures of somatic SVs (Additional file 1: Fig. S18). Fifth, long reads revealed the true structure of SVs. SVs caused by repeat elements, such as germline insertions, may be detected as different types of SVs by short reads (Fig. 2). Sixth, methylation can be analyzed in addition to the genetic variation and somatic mutations (Additional file 1: Fig. S20). These results suggest that long reads are more effective in detecting SVs. Due to short reads having lower sequencing error rates, combining both long and short reads would allow for a more complete landscape of somatic mutations and germline variation to be revealed.

We analyzed germline polymorphisms. Using the long-read sequence data, we could analyze the entire sequences of most insertions and breakpoints of deletions. After excluding SVs within the repeat regions, we classified indels into “deletion events” and “insertion events” by comparing them to the chimpanzee genome. This analysis clearly showed that the majority of insertions were caused by transposons, and *Alu* and LINE strongly contributed to insertions (Fig. 2). In addition to the insertions related to transposons, our analysis identified 106 polymorphic tandem duplication candidates (74 detected from insertions and 32 from intra-chromosomal translocations), 15 polymorphic template sequence insertion candidates, and 15 polymorphic insertions of processed pseudogenes. The numbers of template sequence insertions and insertions of processed pseudogenes were similar to previous studies [34, 45]. These results suggest that transposons play a critical role in indel polymorphism, and the contribution of other types of insertions is rarer. Growing evidence suggests insertions of transposon in human diseases are important [46, 47] and long reads would enable us to discover greater numbers of diseases-associated transposons.

The analysis of deletion breakpoints identified the mechanisms of deletion generation. The application of long reads and the classification of indel events allowed us a more comprehensive analysis than previous short-read and fosmid-based studies [36, 37]. About 80% of NAHR were mediated by SINEs, suggesting that insertions of SINE, which is the most frequent in the insertion events, can cause new deletions. We also found that replication timing and chromatin states were associated with the mutation rate of deletions and insertions. As reported previously, NAHR were significantly enriched in early replicating regions [39], and also in transcription activity regions (Additional file 2: Table S16), suggesting that NAHR frequently occurred in open-chromatin regions. Insertions of *Alu* and LINEs were enriched in quiescent state regions, suggesting that insertions of them are not random.

In the analysis of somatic SVs, long reads enabled us to detect large numbers of somatic SVs. Unlike germline deletions, NAHR was not found in somatic SVs, and the proportions of FoSTeS/MMBIR and NHEJ were higher. Although the numbers of somatic SVs were not similar among samples, the patterns were consistent (Additional file 1: Fig. S21). Analysis of haplotypes revealed complex structures of somatic SVs, and this analysis showed clustered SVs were caused by FoSTeS/MMBIR, NHEJ, and alt-EJ (Additional file 1: Fig. S18). A previous study suggested that clustered SVs can be generated by the breakage-fusion-bridge cycle [41], and our study suggests a possibility that various repair mechanisms are involved in the generation of clustered SVs.

Our analysis detected polymorphic and somatic SVs, revealed true structures of insertions, and inferred the mechanisms for their generation. However, we note that our study has several limitations to be assessed in the future. First, we removed indels in short repeats, as inference of phylogenic status assumes the occurrence of single mutation events between humans and chimpanzees, and this assumption cannot be applied to short repeats. Although mutations in short repeats are considered to be caused by slippage of DNA polymerase [48], further investigations may provide new findings. Second, the average read length of the current study was around 5 kbps, which is sufficient for most SVs. However, longer reads are required to deduce the entire structures of very long insertions, SVs in repetitive regions, and complex haplotype structures. Third, the depth of coverage is not high in the current study. Higher depth would allow the detection of singleton germline SVs and sub-clonal SVs in liver cancers. Fourth, to make our analysis conservative, we set the minimum length of indels to 100 bp, and this study did not analyze indels < 100 bp. We considered that this cutoff value is needed for the current high-error reads (Additional file 1: Fig. S2). However, sequencing technologies and base callers are improving, and in the near future, we should be able to use a smaller cutoff value and identify a larger number of SVs with high accuracy.

## Conclusions

In the present study, we sequenced previously analyzed DNA samples, generated an analysis method, and evaluated the efficacy of long reads in human genetics studies. Our analysis also revealed a complex structure in the cancer genome and sources of variations. We consider long reads to be indispensable for studying genetic variations and somatic mutations, and our method can contribute to future genetic studies.

## Availability and requirements

Project name: Development of advanced data analysis methods for genome sequencing

Home page: https://github.com/afujimoto/CAMPHOR and https://github.com/afujimoto/CAMPHORsomatic

Operating system: Linux

Programming language: python, shell, and perl

Other requirements: samtools (http://www.htslib.org)

License: GPLv3

Any restrictions to use by non-academics: license needed

## Supplementary Information

**Additional file 1:** Supplemental information and Figures. Collection of Supplemental Figures and Supplemental information.

**Additional file 2:** Supplemental Tables. Collection of supplemental Tables.

## Data Availability

Sequencing data have been deposited in the NBDC database in Japan under accession numbers JGAS000180 and JGAD000261 (https://humandbs.biosciencedbc.jp/en/hum0182-v3) [49]. Source code is available from https://github.com/afujimoto/CAMPHOR [50] and https://github.com/afujimoto/CAMPHORsomatic [51, 52].
