# Supplementary material for: Whole-genome sequencing with long reads reveals complex structure and origin of structural variation in human genetic variations and somatic mutations in cancer
Source: Genome Med. 2021 Apr 29;13:65. doi: 10.1186/s13073-021-00883-1 (PMC8082928; doi:10.1186/s13073-021-00883-1)
Supplement: Supplementary file 1 — Additional file 1: Supplemental information and Figures. Collection of Supplemental Figures and Supplemental information. [file 13073_2021_883_MOESM1_ESM.docx]

**Supplemental Information**

*Removal of false positive SVs caused by artificial chimeric reads*

During library preparation, artificial chimeric reads might be generated by random ligation of two or more molecules. Therefore, most artificial chimeric reads should generate SVs that are supported by one read, which can be removed by read number filters. However, we consider that errors caused by sequence duplication of artificial chimeric reads can cause serious errors. During sequencing, MinION sometimes reverses the voltage to try to clear a jam. Therefore, sequencing reads in a pore may be returned to the upper layer of the flowcell, and may be sequenced again (sequence read duplication). If artificial chimeric reads are sequenced twice, false positive SVs supported by multiple reads would be generated. If we selected SVs supported by ≥ 2 reads, we cannot remove them. Therefore, we developed a filter with read information in fastq files.

Fastq files contain the run ID (runid=), channel used for sequencing a read (ch=), and read number (read=) (Additional file 1: Fig. S22). For reads supporting the same SV candidate, we analyzed the read information. We detected reads with same runid, same channel and close read number (difference of read number < 30) or reads with same runid and very close mapping locations, and defined them possible duplicates. From possible duplicates, we selected one read and discarded others. This filtering is effective for removing false positive SVs. In RK067, 446 chromosomal translocations were detected by ≥ 2 reads, but 407 of them were caused by possible duplicated artificial chimeric reads. Filtering improved the accuracy of identification of chromosomal translocations.

This filter has been implemented in CAMPHOR and CAMPHOR somatic. As this filtering needs information on runid, channel ID and read number, which are generally removed in fastq files in database, we could not use this filter for the benchmarking with NA19240.

**Supplemental Figures**

**Fig. S1: Pattern of SVs. (a-e) Expected read pattern of SVs.** Blue indicates inserted sequences. **(f-j) Examples of SVs.** Ranges of deletions are shown in red rectangles. Breakpoints of the inversion and insertions are shown in arrows. Reads were shown with IGV[1].

**Fig. S2: Summary of sequence reads.** (**a) Distribution of read lengths in this study. (b) Distribution of average base quality of mapped and unmapped reads. (c) Proportions of unaligned bases in mapped reads. (d) Distribution of mapping quality by minimap2 software**[2] **(e) Distribution of error rates.**

**Fig. S3: Length and frequency spectrum of germline indels. (a) Length of indels. (b) Frequency spectrum of deletions.** Deletions overlapping coding regions (CDS) had higher number of rare variants (Number of doubleton deletions in CDS; other deletions in CDS). **(c) Frequency spectrum of insertions.** All; all indels, CDS; indels overlapping coding regions, non-SR; indels not in short repeat regions.

**Fig. S4: Result of benchmarking using NA19240 [3].** Numbers of indels detected by each caller and gold standard set were compared. Common; detected in gold-standard SV calls and each caller, Gold-standard SV call only; detected only in gold-standard SV calls, and Caller only; detected only in each caller. SVs were classified based on repeat information of regions. SVs in tandem repeat regions, self-chain regions, regions in both repeats, and non-repeat region were evaluated separately.

**Fig. S5: Flow of inferring mechanisms of indels.** We extracted sequences around insertions and deletions from each read (insertion; inserted sequence and 500bp of flanking sequences, deletion; 500bp of flanking sequences). The extracted sequences were aligned with MAFFT software[4] and consensus sequences were generated for each insertions and deletions. The consensus sequences were then mapped to the human reference genome and locations of insertions and deletions were redefined. The inserted sequences and deleted sequences were analyzed by the Tandem Repeat Finder[5] and RepetMakser[6] software and sequences that were not caused by short repeats were gathered. Their lengths were compared with the those in the Chimpanzee genome and the phylogenetic status was estimated. Based on the phylogenetic status, we inferred events that caused insertions and deletions (Fig. S7).

**Fig. S6: Proportion of short repeats in inserted and deleted sequences.** Deleted and inserted sequences were analyzed by RepeatMasker and Tandem Repeat Finder software, and proportions of repeats by Tandem Repeat Finder and”Simple_repeat", "Low_complexity" and "Satellite” by RepeatMasker were calculated.

**Fig. S7: Inferring true events of indel generation.** Events that generated indels were inferred by comparison with the Chimpanzee genome sequence. **(a) Pattern of deletions caused by an insertion event. (b) Pattern of deletions caused by a deletion event. (c) Pattern of insertions caused by an insertion event. (d) Pattern of insertions caused by a deletion event.**

**Fig. S8: Number of insertion events in each repeat.** Number of insertion events in each location was counted for each repeat family. 5’ regions have slightly lower numbers of insertions in *Alu* families, indicating that most *Alu* insertions are full-length and some parts lack 5’ region. In LINE L1HS, more than half of insertions were not full length and only 3’ parts were inserted in the rest of the insertions. This is consistent with the mechanisms of insertions.

**Fig. S9: Strands of combination of two SINEs.** Our analysis detected insertion events covered by two SINEs. Although we could not find any characteristic pattern in the families of the combinations, we found that combinations of same strand were significantly larger than these of different strands (Fisher’s exact test).

**Fig. S10: Expression level of candidate origins of processed pseudogenes.** Gene expression level of the 15 origin genes and other genes were compared in GTEx expression data[7]. All comparisons were statistically significant after Bonferroni correction (Wilcoxon signed-rank test) (Additional file 2: Table S10).

**Fig. S11: Consensus sequence generation of flanking region of deletions and inference of mechanism. (a) Procedure of data analysis.** We extracted sequences of flanking regions of deletions (±500bp from the breakpoints) from each read and assembled them with MAFFT [4] software. Consensus sequences were decided by majority. The consensus sequences were aligned to the reference genome sequences, and homology and insertion at breakpoints were analyzed. **(b) Examples of patterns.** Examples of NHEJ, alt-EJ and FoSTeS/MMBIR candidate are shown. This analysis was performed according to previous studies [8][9].

**Fig. S12: Decision algorithm and number of candidate mechanism of germline deletions. (a) This decision-making algorithm is based on Kidd et al. (2010) and Yang et al. (2013)** [8][9]**. (b) Comparison with Yang et al. (2013)** [9]**.** The numbers of each type are similar in the two studies. In addition to their result, our long-reads study detected NARH events.

**Fig. S13: Comparison of deletion lengths. (a) Distribution of deletion lengths. (b) Result of the comparison of length between NAHR, alt-EJ, FoSTeS/MMBIR and NHEJ.** Comparisons were done by Mann-Whitney U test. Yellow indicates *p-values* significant after Bonferroni correction (n = 6).

**Fig. S14: Inserted sequence of NHEJ.** **(a) Length of insertion at breakpoint of NHEJ. (b) Base composition of 1bp insertions.** The proportions were not significantly different from that by chance (*p-value* = 0.89, Chi-square test).

**Fig. S15: Insertion sequence of FoSTeS/MMBIR candidates. (a) Length of insertion at breakpoint of FoSTeS/MMBIR. (b) Mapping results and electropherograms of the Sanger sequencing.** Upper panel shows output of web-BLAT (https://genome.ucsc.edu/cgi-bin/hgBlat). Mapping result of the consensus sequence and the reference sequence are shown. Blue and black indicates aligned and unaligned bases. Black characters in upper (consensus) and lower (reference) sequences show that an insertion and a deletion co-occurred. Inserted sequences in the electropherogram are indicated by red rectangles.

**Fig. S16: Location of breakpoints in SINE in breakpoints. (a) Analysis of strands of right SINE and left SINE.** *P-values* were calculated by Fisher’s exact test. **(b) Correlation of location in SINE.** Correlation was examined by Pearson's product moment correlation coefficient.

**Fig. S17: Comparison between short-reads and long-reads in cancer SVs. (a) Comparison of VAF between common SVs (detected by short-reads and long-reads) and Short-reads only (detected only by short-reads).** *P-value* was obtained by Mann-Whitney U test. **(b) Comparison of VAF of commonly identified SVs.** All types of SVs showed high correlations (Chromosomal translocation; *r* = 0.55, *p-value* = 2.6x10^-8^, Deletion; *r* = 0.75, *p-value* < 2.2x10^-16^, Inversion; *r* = 0.76, *p-value* < 2.2x10^-16^, and Intra-chromosomal translocation; *r* = 0.69, *p-value* < 2.2x10^-16^ by Pearson's product moment correlation coefficient). **(c) Number of SVs between short-reads**[10] **and long-reads (current study).** The SV numbers were significantly correlated among samples (*r* = 0.96, *p-value* = 3.2x10^-6^).

**Fig. S18: Examples of haplotype structure of somatic SVs in liver cancer.** Reads with multiple SVs and location of SVs on the reads are shown. Upper line shows read and other lines indicate mapped genomic segments. Gray indicates reverse strand of genome. Type of breakpoint is shown below each breakpoint.

**Fig. S19: HBV integration to *MLL4* gene. (a) Insertion in *MLL4* gene in RK014. (b) Insertion in *MLL4* gene in RK147.** Long-reads revealed entire structure of the integrations. Reads were shown with IGV [1]. **(c) PCR validation of the integrations.** B; blood sample, C; cancer sample.

**Fig. S20: Methylation analysis of *ALB* and *TERT* prompter regions. (a) Methylation rate of *ALB* promoter.** Reads were extracted from WGS data of blood and liver cancer samples. *ALB* is known to be a highly expressed gene in liver, therefore, methylation rate of *ALB* gene promoter would be different between liver cancer and blood samples. As expected, methylation rates of *ALB* promoter were quite different between cancer and blood, and liver cancers have lower methylation rates, suggesting that tombo functioned successfully in the methylation analysis. **(b) Methylation rate of *TERT* promoter.** Liver cancer samples with and without mutations in *TERT* promoter were compared. In RK014, RK020, RK067, RK085 and RK167 had mutation in our previous study[10] and were used as mutated samples. Methylation rates were estimated for each CpG site with tombo software. M; samples with mutation, N; samples without mutation. Methylation rates were compared in 400bp bins using t-test.

**Fig. S21: Number of somatic SVs in each sample. (a) Chromosomal translocations. (b) Deletions. (c) Inversion. (d) Intra-chromosomal translocation.**

*
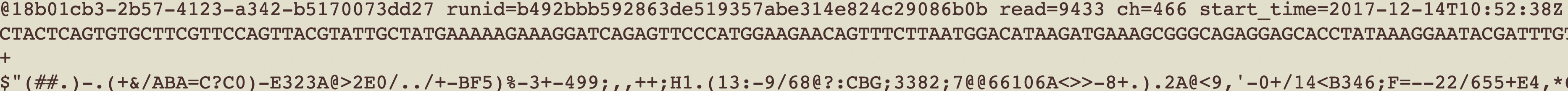
*

**Fig. S22: Example of read information in fastq file.** For filtering possible duplicated artificial chimeric reads, we used run ID (runid=), the channel used for sequencing reads (ch=), and read number (read=)

**References**

1. Robinson JT, Thorvaldsdóttir H, Winckler W, Guttman M, Lander ES, Getz G, et al. Integrative genomics viewer. Nat. Biotechnol. 2011.

2. Li H. Minimap2: Pairwise alignment for nucleotide sequences. Bioinformatics. 2018;

3. De Coster W, De Rijk P, De Roeck A, De Pooter T, D’Hert S, Strazisar M, et al. Structural variants identified by Oxford Nanopore PromethION sequencing of the human genome. Genome Res. 2019;29:1178–87.

4. Katoh K. MAFFT: a novel method for rapid multiple sequence alignment based on fast Fourier transform. Nucleic Acids Res. 2002;

5. Benson G. Tandem repeats finder: A program to analyze DNA sequences. Nucleic Acids Res. 1999;

6. Nishimura D. RepeatMasker. Biotech Softw Internet Rep. 2000;

7. Melé M, Ferreira PG, Reverter F, DeLuca DS, Monlong J, Sammeth M, et al. The human transcriptome across tissues and individuals. Science (80- ). 2015;

8. Kidd JM, Graves T, Newman TL, Fulton R, Hayden HS, Malig M, et al. A human genome structural variation sequencing resource reveals insights into mutational mechanisms. Cell. 2010;

9. Yang L, Luquette LJ, Gehlenborg N, Xi R, Haseley PS, Hsieh CH, et al. Diverse mechanisms of somatic structural variations in human cancer genomes. Cell [Internet]. Elsevier Inc.; 2013;153:919–29. Available from: http://dx.doi.org/10.1016/j.cell.2013.04.010

10. Fujimoto A, Furuta M, Totoki Y, Tsunoda T, Kato M, Shiraishi Y, et al. Whole-genome mutational landscape and characterization of noncoding and structural mutations in liver cancer. Nat Genet. 2016;48:500–9.
